# One-Haptic Fixation of Posterior Chamber Intraocular Lenses without Scleral Flaps

**DOI:** 10.1155/2012/891839

**Published:** 2012-08-05

**Authors:** Ashraf I. Moawad, Asaad A. Ghanem

**Affiliations:** Ophthalmic Center, Faculty of Medicine, Mansoura University, P.O. Box 35516, Mansoura, Egypt

## Abstract

*Purpose*. To assess visual results and complications of a modified technique of posterior chamber intraocular lenses (PC IOLs) in aphakic eyes without scleral flaps. *Methods*. Modified one-haptic scleral fixation was performed in one eye each of 25 patients with aphakia and insufficient capsule support. Follow-up period was six months. Outcome measures included best-corrected visual acuity, intraocular pressure (IOP), and postoperative complications. *Results*. The study included 15 males and 10 females. The preoperative best-corrected visual acuity (BCVA) ranged from 5/60 to 6/9. The operation time ranged from 25 to 45 minutes (mean 35.25 ± 5.34 min). Anterior vitrectomy was performed in 12 cases (48%). There was no major IOL decentration. The final BCVA ranged from 6/36 to 6/9. Seven cases (28%) showed postoperative glaucoma, five cases (20%) had temporary hypotony, and hyphema in 2 eyes (8%). No cases of suture erosion, postoperative endophthalmitis, retinal detachment, or IOL dislocation were detected. *Conclusion*. This technique of one-haptic scleral fixation of posterior chamber IOLs is a good choice in presence of insufficient capsule support. It reduces the operation time, achieves the IOL stability, and minimizes postoperative suture-related complications.

## 1. Introduction 

 There are three methods to implant an intraocular lens (IOL) in presence of inadequate capsule support: anterior chamber IOL implantation, iris-sutured posterior chamber IOL implantation, and transscleral sutured posterior chamber IOL implantation [1]. Transscleral sutured posterior chamber IOLs have been shown to be safe and effective for optical rehabilitation of aphakic eyes in which capsule support is inadequate [2].

 Although transscleral sutured posterior chamber IOL is technically difficult, it avoids complications with anterior chamber IOL implantation, such as corneal endothelial decompensation, uveitis-glaucoma-hyphema syndrome, and cystoid macular edema [3]. Transcleral sutured posterior chamber IOLs, when placed properly, do not contact the iris and therefore reduce the risk for complications associated with iris-sutured IOLs [4].

Tilting and decentration of transscleral sutured posterior chamber IOL can occur [5]. Also, breakage of the polypropylene sutures is the most common late postoperative complication necessitating a reoperation [6]. Minimally traumatic surgery and stable IOL placement are imperative for good functional and anatomic results [7].

Scharioth et al. [8] Fixation of PC IOL hepatics in a limbus-parallel scleral tunnel provided exact centration and axial stability of the IOL and prevented distortion and subluxation in most case.

The aim of the present study is to evaluate the visual results and complications of one-haptic scleral fixation of posterior chamber intraocular lenses in aphakic eyes with inadequate capsule support without scleral flaps.

## 2. Patients and Methods

### 2.1. Study Design

 This was a prospective study. After explaining the details of the study, we obtained written informed consent from all patients before enrollment. The study was approved by Mansoura University Hospital trust ethics committee and was carried out in accordance with the Decleration of Helsinki (1989) of the world medical association.

### 2.2. Patients

 This prospective study was conducted on 25 eyes of 25 patients attending Ophthalmic Center, Mansoura university, Egypt. The causes of aphakia were accidental rupture of the posterior capsule during extracapsular cataract extraction for senile cataract in 15 cases, traumatic cataract with posterior capsule tears without primary implantation in 10 cases.

 Inclusion criteria were age between 45 years and 63 years, and patient motivation (i.e., desire to no longer wear of spectacle or contact lens). Exclusion criteria included more than 1.00 diopter of corneal astigmatism, central corneal scars, history of glaucoma and posterior segment disorders, previous corneal or intraocular surgery, abnormal iris, and history of ocular inflammation.

 All patients involved in the study were subjected to the following ophthalmic examinations: distance visual acuity assessment (unaided and best corrected), slit-lamp biomicroscopy of the anterior segment, intraocular pressure measurement (IOP) by Goldmann applanation tonometry, A/B-scan immersion ultrasonography, keratometry, and biometry. SKR II formula was used with reduction of IOL power of 0.5 D to compensate for the more anterior IOL location in the ciliary sulcus instead of the capsular bag using stratus OCT (model 3000, software version 4.4; Carl Zeiss Meditec, Dublin, CA, USA) by circular 360° OCT scans were obtained. In addition, clinical data regarding age, gender, systemic medication, and previous ocular surgery were recorded. 

### 2.3. Surgical Technique

The preoperative pharmacologic preparation in all patients was cyclopentolate 1% and phenylephrine 2.5% 30 minutes before surgery.

### 2.4. Technique of Surgery (Figure 1)

- Limbal-based conjunctival flap was created opposite the site of the capsular remnants and half thickness 3 mm scleral grooves 1.0 mm behind the limbus were constructed using a crescent blade.
- Double-armed 10–0 polypropylene (Prolene) sutures on straight needle were used (STC-610/0 Prolene 16 mm, 23 cm; Ethicon New Jersey, USA). The straight needle was held by the needle holder and passed through the scleral tunnel behind the iris until it was seen in the pupillary area (a).
- The needle directed toward the opposite limbus and extracted through the cornea (b) and the thread is cut by a scissor. A stepped corneal incision according to the site of capsular remnant was done with a 3-mm Keratome, and the thread is pulled externally through it (c).
- Anterior vitrectomy was performed in cases with vitreous in the anterior segment through the corneal incision, and an ophthalmic viscosurgical device (sodium hyaluronate 1% [Healon]) was injected into the eye, filling the retropupillary space and the anterior chamber.
- A single-piece biconvex polymethylmethacrylate (PMMA) posterior chamber IOLs (Corneal-CP100, Paris, France) were used. The IOLs have a 118.5 A-constant, a 13.80 mm overall diameter, 7.0 mm optic, and 2 holes on each haptic.
- The IOL was implanted with one haptic supported over the capsular remnant and the prolene thread was, passed as a loop through the eyelet of the other haptic and tightened around it in double suturing manner (d).
- The haptic was drawn into the sulcus by pulling on the externalized suture. The externalized suture was secured by taking a bite of sclera in the bed of the scleral tunnel and tying the suture to itself (e). It was trimmed slightly long to allow it to lie flat with knot buried under the scleral tunnel.
- The corneal incision was enlarged, and the IOL was implanted with gentle pulling on the prolene suture.
- The corneal incision was closed using interrupted 10/0 monofilament nylon suture, and the conjunctive was closed with 7.0 vicryl.

Postoperatively, all eyes received topical drops containing 0.3% tobramycin and 0.1% dexamethazone four times a day for first week and then tapered off within four weeks.

Patients were examined postoperatively daily during first week, then weekly during first month, then every month for six months for best-corrected visual acuity (BCVA), intraocular pressure measurement, postoperative complications such as cystoid macular oedema, retinal detachment, and vitreous hemorrhage.

### 2.5. Statistical Analysis

 Statistical analysis was performed using SAS software (SAS Institute, Inc.). All analyses were adjusted from the 6-month visits. The statistical analyses were adjusted using Benforroni correction. 

 The student *t* test was used for parametric variables, and the chi-square test for categorical variables. The data fall in normal disturbation. Results are presented as the mean ± standard deviation. A *P* value < 0.05 was considered to be significant. 

## 3. Results

Twenty five eyes of 25 patients ages ranged from 45 to 63 years (mean 52 ± 6.45) were enrolled in the present study. Males were 15 (60%) patients, and females were 10 (40%) patients. 

The duration of the operation ranged from 25 to 45 minutes (mean 35.25 ± 5.34 min). Anterior vitrectomy was performed in 12 cases (48%). There was no major IOL decentration or tilt detected in any case.

 The final BCVA ranged from 6/36 to 6/9. Seven cases (28%) showed postoperative glaucoma (four cases responded to topical antiglaucoma medications, and the other three cases underwent subscleral trabeculectomy with intraoperative mitomycin C application.), five cases (20%) had temporary hypotony (four cases with overfiltration and one with wound leak), and hyphema in 2 eyes (8%). Corneal oedema developed in 5 eyes (20%) and resolved before the end of follow-up period. No cases of suture erosion, postoperative endophthalmitis, retinal detachment, or IOL dislocation were detected. 

 The postoperative UCVA was measured on the first postoperative day, after one month, three months, and six months. The results were as follows: on the first postoperative day, the UCVA ranged from 2/60 to 6/24. After the first postoperative month, the UCVA ranged from 4/60 to 6/18. After the third postoperative month, the UCVA ranged from 5/60 to 6/18. At six months postoperatively the UCVA ranged from 6/60 to 6/18. The BCVA postoperatively at six months ranged from 6/36 to 6/18.

 Postoperative iridocyclitis was observed in 3 eyes (12%) that needed the use of atropine sulphate 1% and frequent topical corticosteroids (prednisolone acetate 1%). Other postoperative complications that included suture erosion, postoperative endophthalmitis, retinal detachment, irregular astigmatism, or IOL dislocation were not detected. The posterior chamber IOL remained well centered and without tilt in all eyes. 

## 4. Discussion

 The ciliary sulcus is a slightly vascular area, and IOL placement in this anatomic area is more or less stable because of the surrounding structures [9]. Different techniques of transscleral suturing of posterior chamber IOLs have played an important role in the visual rehabilitation of aphakic eyes lacking capsule support [7]. A disadvantage of ab-interno technique is that during penetration, with the needle tip obscured by the iris, the direction of the needle and penetrating site are sometimes difficult to control [10]. Ab-externo transsclerally sutured IOL assure correct placement of the anchoring sutures and thus the placement of the lens haptic [11].

Various techniques of fixation of the suture to the IOL and sclera have been developed. However, there have been problems with techniques of PC IOL suture fixation that can result in a suboptimal visual result. In theory, iris chafing and uveitis can result from free suture ends rubbing on the ciliary body and iris. These free suture ends may also unravel, resulting in IOL dislocation [1]. Both Eryilidirim [1] and Grigorian et al. [4] describe a modified technique in which after the suture loop is passed through the eyelet, it is looped around the end of the haptic. When tightened, this loop is fixated at the eyelet on the free side of the haptic.

 In the present study, the modified technique of scleral fixation of posterior chamber IOL depends on supporting the IOL by placing one haptic over the capsule remnants and suturing the other haptic to the sclera in the opposite side. Double knot fixation of the haptic has the advantages of good haptic fixation and minimizes the risk for slippage. These advantages are also described by Chen et al. [12]. Suturing the IOL in one side only and enlarging the corneal incision when the IOL is ready to be inserted minimize the duration where the globe is open, thus reducing the incidence of expulsive choroidal heamorrhage, which is considered to be related to increased surgical time and manipulation as described by Chu and Green [13]. Passing the prolene suture through the scleral groove and the holes without interruption keeps the integrity of the suture. Making scleral tunnel rather than scleral flap reduces time of surgery and avoids unnecessary trauma to the eye. This step is also described by Monteiro et al. [14].

 This technique aimed at improving the visual acuity outcome by reducing the IOL tilt and decentration. Also, It tried to shorten the operation time, reduce the complications, and give good centration. 

The present study found postoperative iridocyclitis in 3 eyes (12%) that needed the use of atropine sulphate 1% and frequent topical corticosteroids (prednisolone acetate 1%). Other postoperative complications that included suture erosion, postoperative endophthalmitis, retinal detachment, irregular astigmatism, or IOL dislocation were not detected. 

 As regards postoperative complications, seven cases showed postoperative glaucoma which consisted with Monteiro et al. [14]. Four cases responded to topical antiglaucoma medications and the other three cases underwent subsclerat trabeculectomy with intraoperative mitomycin C application.

 Five cases had temporary hypotony, and hyphema in two eyes. Postoperative hyphema resolved by conservative management. Transient corneal edema occurred due to high or low intraocular pressure, vitreous loss, fluid stream during anterior vitrectomy, or postoperative anterior uveitis. This corneal edema resolved during the follow-up period. These results are similar to those recorded by Lin and Tseng [15].

Han and Chu [16] described an intraocular lens (IOL) fixation technique that combines suture-in-needle and scleral tunnel techniques. A 10–0 polypropylene suture is inserted into the barrel of a 27-gauge sharp needle to tie the IOL haptic, and scleral tunnels are created to bury the knots for transscleral IOL fixation. The modification of the traditional scleral fixation technique simplifies the creation of a scleral covering and decreases harmful manipulations of the needle passing through the vitreous cavity.

López-Guajardo and Benítez-Herreros [17] found that sutureless intrascleral posterior chamber intraocular lens fixation is, in our experience, a safe technique that allows locating the IOL in the posterior chamber when no capsular support is present. Visual and refractive outcomes were satisfactory.

 This study is limited due to small number of cases and requires a long-term followup.

In conclusion, one-loop scleral fixation posterior chamber IOL reduced the operation time, achieved good centration and stability of the IOL, and minimised postoperative suture-related complications.

## Figures and Tables

**Figure 1:**
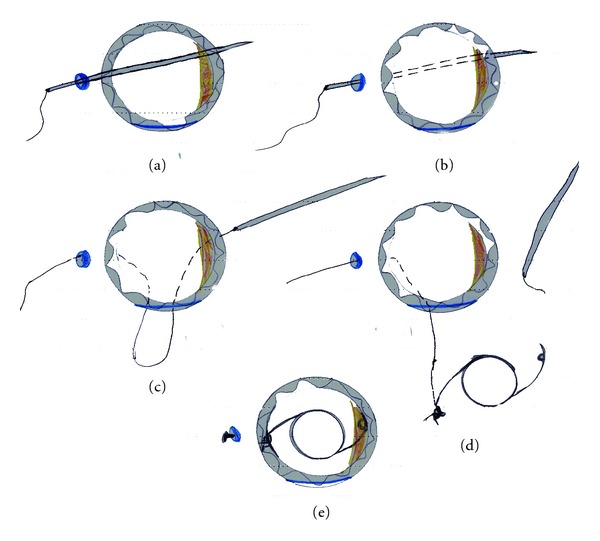
Steps of one-haptic scleral fixation posterior chamber IOL. (a) The needle is inserted into the anterior chamber. (b) The needle is directed toward the opposite limbus. (c) The thread is pulled externally through corneal incision. (d) The suture ends are tied to the haptic. (e) The suture-fixated IOL is placed after the external ends are tied.
